# Review and Updates on Systemic Mastocytosis and Related Entities

**DOI:** 10.3390/cancers15235626

**Published:** 2023-11-28

**Authors:** Julie Y. Li, Christopher B. Ryder, Hailing Zhang, Samuel G. Cockey, Elizabeth Hyjek, Lynn C. Moscinski, Elizabeth Sagatys, Jinming Song

**Affiliations:** 1 Department of Pathology and Laboratory Medicine, H. Lee Moffitt Cancer Center, Tampa, FL 33612, USA; 2 Morsani College of Medicine, University of South Florida Health, Tampa, FL 33602, USA

**Keywords:** systemic mastocytosis (SM), mast cell activation syndrome (MCAS), hereditary alpha-tryptasemia (HαT or HAT), classification, diagnosis, molecular, *KIT*, treatment, the International Consensus Classification (2022 ICC), WHO 5th Edition Classification of Haematolymphoid Tumours (WHO 5th edition)

## Abstract

**Simple Summary:**

Mast cells are specialized immune cells that mediate allergic and anaphylactic reactions, among other immunologic functions. The accumulation of abnormal mast cells can give rise to various disorders that range in severity of symptoms and in impact on life expectancy. This review outlines the range of mast cell disorders, with a focus on systemic mastocytosis, a rare clonal neoplastic mast cell proliferation in various tissues, often associated with *KIT* mutations that cause uncontrolled mast cell growth. Emphasis is placed on the latest diagnostic criteria and approaches because accurate diagnosis and classification are crucial for effective management and treatment.

**Abstract:**

Mast cell disorders range from benign proliferations to systemic diseases that cause anaphylaxis and other diverse symptoms to mast cell neoplasms with varied clinical outcomes. Mastocytosis is the pathologic process of the accumulation of abnormal mast cells in different organs, mostly driven by *KIT* mutations, and can present as cutaneous mastocytosis, systemic mastocytosis (SM), and mast cell sarcoma. The WHO 5th edition classification divides systemic mastocytosis into bone marrow mastocytosis, indolent systemic mastocytosis, smoldering systemic mastocytosis, aggressive systemic mastocytosis, systemic mastocytosis with an associated hematologic neoplasm, and mast cell leukemia. The new ICC classifies SM slightly differently. The diagnosis of SM requires the integration of bone marrow morphologic, immunophenotypic, and molecular findings, as well as clinical signs and symptoms. Moreover, understanding the wide range of clinical presentations for patients with mast cell disorders is necessary for accurate and timely diagnosis. This review provides an updated overview of mast cell disorders, with a special emphasis on SM, including the latest approaches to diagnosis, prognostic stratification, and management of this rare disease.

## 1. Introduction

Mast cells (MCs) are immune cells which are responsible for several immunologic functions, most notably allergic reactions, by releasing stored mediators. Mast cell disorders result from the overgrowth of and/or inappropriate mediator release by MCs, leading to symptoms in various organs. This review explores the intricate world of MCs and delves into the complexities of related disorders, including mast cell activation syndrome (MCAS), hereditary alpha-tryptasemia (HαT/HAT), and mastocytosis. MCAS, a condition marked by severe MC activation, leads to diverse multisystem inflammatory and allergic reactions [1]. Concurrently, HαT, an autosomal dominant disorder resulting from genetic alterations, elevates basal serum tryptase levels, intensifying symptoms, particularly in mastocytosis patients [2]. Mastocytosis is a rare clonal hematopoietic disorder characterized by proliferation of neoplastic MCs in different organs [3].

As a recognition of its distinct biology, mastocytosis was initially distinguished from myeloproliferative neoplasms (MPN) in the 2001 World Health Organization (WHO) classification [4,5], and it was integrated into MPN by the 2008 WHO [6]. In 2016, mastocytosis was once again separated from MPN, delineating three subtypes: cutaneous mastocytosis (CM), systemic mastocytosis (SM), and mast cell sarcoma (MCS) [7]. Based on the proposal of diagnostic criteria and classification of MC disorders by the Europe (EU)/USA consensus group in 2021 [8], these criteria were subsequently updated in both the 2022 International Consensus Classification (ICC) [9] and the WHO 5th edition classification of haematolymphoid tumours [10]. Each of these classifications incorporates the latest understanding of this family of diseases, but subtle differences exist and will be highlighted herein.

Whereas CM is limited to skin and is predominantly a pediatric disorder that often carries a good prognosis, SM involves internal organs with or without cutaneous involvement, is more common in adults, and exhibits a variable prognosis [11]. SM is categorized into non-advanced variants (bone marrow mastocytosis or BMM, indolent systemic mastocytosis or ISM, and smoldering systemic mastocytosis or SSM) and advanced variants (aggressive systemic mastocytosis or ASM, systemic mastocytosis with an associated hematological neoplasm or SM-AHN, and mast cell leukemia or MCL). Therapies may be directed toward symptom management or eradication of the neoplastic MCs, with multikinase inhibitors targeting *KIT* D816V and other vital signaling molecules emerging as novel options for advanced SM.

Given the variety of MC disorders and their often non-specific and overlapping presentations, their diagnosis and management requires a high degree of clinical suspicion as well as thorough clinical and pathological evaluation that includes state-of-the art diagnostics. This article provides a comprehensive overview of SM and its overlapping disorders MCAS and HαT, focusing on its multidisciplinary dimensions and the intricate challenges it poses in daily medical practice. Emphasizing the current state of diagnosis and classification, it incorporates both the WHO 5th Edition and the 2022 ICC schema. Additionally, the review explores molecular updates and targeted therapies in adult SM, illuminating the evolving landscape of mastocytosis research and resulting clinical interventions.

## 2. Overview

### 2.1. Normal Mast Cells

Mast cells (MCs) derive from CD34+/KIT+ pluripotent hematopoietic progenitors in the bone marrow. MC precursors traffic to and undergo terminal maturation in various vascularized tissues; mature MCs are normally absent from peripheral blood [12]. MCs play an important role in inflammatory reactions through the release of mediators stored within their granules, a process that can be triggered by both innate and adaptive immune mechanisms. MCs are characterized by high expression levels of two surface receptors: the high-affinity IgE receptor (FcεRI) and CD117 (KIT), a receptor for stem cell factor (SCF). Crosslinking of FcεRI to the Fc region of circulating IgE triggers mast cell degranulation and the release of inflammatory and vasoactive mediators [13,14,15]. KIT (CD117) is a Type III receptor tyrosine kinase activated by the binding of SCF, leading to dimerization and autophosphorylation, and subsequent downstream signaling that promotes MC differentiation, proliferation, maturation, and degranulation [14,16,17,18]. The integration of signaling pathways initiated by KIT and FcϵRI synergistically enhances mediator release and contributes to clinical symptoms. MC-derived mediators include histamine, tryptases, prostaglandin D2 (PGD2), platelet-activating factor, multifunctional cytokines (TNF-α, TNF-β, SCF, IL-1, IL-2, IL-3, IL-4, IL-5, IL-6, IL-9, IL-10, IL-13, and GM-CSF), leukotrienes (LTC4, LTD4, and LTE4), and chemokines (IL-8, MCP-1, and MIP-1α [19,20,21].

Microscopically, MCs tend to concentrate near small blood vessels and at the periphery of lymphoid nodules or aggregates [22,23]. In normal bone marrow, MCs are relatively few (<1%) with a perisinusoidal or paratrabecular distribution [22]. MCs are round mononuclear cells with round nuclei, inconspicuous nucleoli, and densely packed metachromatic granules that obscure the nucleus. On H&E-stained tissue sections, MCs are often easily recognized by the distinct cytoplasmic metachromatic granules. However, at times, they may not be well-visualized under the microscope, possibly due to MC granules leaching out during the tissue-processing [24]. Normal mature MCs present a distinct immunophenotype with high CD117, intermediate CD33 (similar to monocytes), CD9 and CD71, low CD11b and CD38, and absent HLA-DR, CD34, and CD123 expression [25].

### 2.2. Mast Cell Related Disorders

MC hyperplasia describes an increase in non-clonal, non-neoplastic MCs. This reactive process can be seen in myeloid neoplasia, lymphocytic lymphoma, marrow hypoplasia/aplasia, administration of SCF, and a variety of toxic or inflammatory exposures. These MCs demonstrate normal morphology and immunophenotype [21].

MCAS is a syndrome associated with severe, acute MC activation and excessive mediator release, resulting in multisystem inflammatory and allergic reactions [1]. MCAS occurs primarily in patients with IgE-dependent allergies, mastocytosis, and HαT [21]. The patients often present with non-specific symptoms, and clinical courses range from indolent to highly aggressive, which makes diagnosis challenging [26]. MCAS is not considered a subtype of mastocytosis, nor is it necessarily a premalignant condition [27]. Not surprisingly, however, patients with SM show higher rates of MCAS and severe anaphylactic reactions than do healthy controls [28,29]. Based on the etiology, MCAS can be divided into five variants: (1) primary/monoclonal MCAS (MMAS), where *KIT*-mutated, clonal MCs are detected, and it may either fulfill the criteria for mastocytosis (SM or CM) or present with only two minor SM criteria; (2) secondary MCAS where the disorders is triggered by an underlying IgE-dependent allergy or other immunologic disorder; (3) HαT MCAS; (4) combined MCAS where more than one of the above pathologies co-exist; and 5) idiopathic MCAS, in which none of the above causative triggers can be identified [30,31,32,33] (Table 1).

There are no MCAS diagnostic criteria established by the WHO 5th edition and 2022 ICC. Proposed consensus diagnostic criteria for MCAS [21,30,34] involve meeting all three criteria: (1) symptoms of recurrent MC activation affecting two or more organ systems; (2) biochemical evidence of MC activation (preferred marker—increase in serum tryptase level from baseline by 20% plus 2 ng/mL); and (3) response of symptoms to therapy with MC-stabilizing agents or with drugs directed against MC mediator production, release, or downstream effects. Mediators can be measured in a variety of biological fluids (serum, plasma, or urine) [30], with tryptase and PGD2 being among the more specific biomarkers for MC activity. A reliable indicator of disease activity is the rapid increase in serum tryptase levels from baseline. [35,36,37,38].

Hereditary alpha-tryptasemia (HαT or HAT) is an autosomal dominant condition resulting from extra copies of the *TPSAB1* gene, which codes for α-tryptase [39]. HαT affects approximately 6% of the general population, accounting for 90% of cases with elevated basal serum tryptase (BST) levels [40]. Most patients with HαT are asymptomatic, while others may experience symptoms such as cutaneous flushing, pruritus, dysautonomia, gastrointestinal (GI) issues, chronic pain, and joint hypermobility. Recent studies have demonstrated that HαT significantly affects the severity of various MC-related disorders, including SM, idiopathic anaphylaxis, and venom allergy. HαT is more prevalent in patients with SM (17–18%) compared to healthy controls (5–7%), especially those with ISM and SSM [41,42]. Concurrent HαT predisposes mastocytosis patients to Hymenoptera venom hypersensitivity reactions and severe MC mediator-related cardiovascular symptoms [41,43,44].

Mastocytosis, a rare disorder (estimated 1 per 100,000) [45], is defined by abnormal proliferation of clonal MCs in different tissues, including skin, bone marrow (BM), GI tract, liver, and/or spleen [9]. It is frequently associated with somatic gain-of function mutations in *KIT*, leading to constitutive KIT activation independent of its ligand SCF [18,46]. Based on the pathology, clinical presentations, and organ involvement, mastocytosis is divided into three major groups by both the 2022 ICC and WHO 5th edition classifications (Table 2) [10,33,47,48]. CM, typically diagnosed in pediatric patients, is limited to the skin and generally carries a good prognosis, as the skin lesions usually improve or resolve during puberty. CM can be further divided into urticaria pigmentosa (UP, also termed maculopapular CM), diffuse CM, and cutaneous mastocytoma. Based on MC burden, organ involvement, and SM-related organ damage, SM is further categorized into five distinct subtypes: ISM, SSM, ASM, SM-AHN, and MCL [2]. ISM and SSM are non-advanced SM variants with an indolent clinical course, while ASM, SM-AHN, and MCL are collectively referred to as advanced SM based on evidence of organ dysfunction and/or damage by MC infiltration. MCS is a highly aggressive, destructive proliferation of neoplastic MCs that tends to metastasize and culminate in an MCL-like picture.

## 3. Pathogenesis of Systemic Mastocytosis (SM)

**Figure 1:**
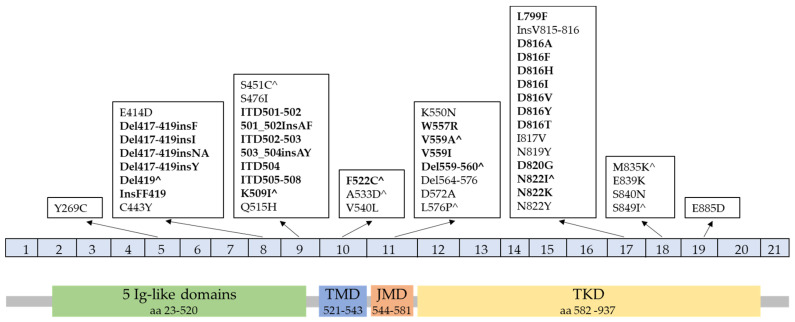
Representative illustration of *KIT* gene (**top**) and protein (**bottom**) with mastocytosis-associated mutations. Listed are all reported *KIT* variants identified in mastocytosis (modified from [8]). Bolded mutations are known to constitutively activate KIT, qualifying as oncogenic variants and a minor criterion for SM. ^ Denotes *KIT* mutations found in germline configuration in familial cases, mainly in CM. Variants are arranged by affected exons of *KIT* cDNA, which align to KIT protein functional domains. Ig = Immunoglobulin, JM = juxtamembrane domain, TKD = tyrosine kinase domain, and TM = transmembrane domain.

SM is the most common form of mastocytosis in adults, and it tends to persist and progress [51]. The proliferation of neoplastic MCs is usually driven by *KIT* mutations that activate downstream pathways including PI3K, STAT5, NF-κB, mTORC2, and PKCδ [52]. KIT (also known as c-KIT), a transmembrane receptor encoded by *KIT* on chromosome 4q11~12, consists of 976 amino acids, divided into extracellular domains (aa 23–520) characterized by five immunoglobulin (Ig)-like domains (aa 23–520), a transmembrane (TM) region (aa 521–543), and an intracellular tail, including a juxtamembrane (JM) domain (aa 544–581) and a tyrosine kinase (TK) domain (aa 582–937) (see Figure 1, bottom) [16]. The *KIT* D816V mutation (exon 17) is frequently detected (>90%) in adult SM. Other less common mutations at codon 816 of *KIT*, including D816F, D816Y, D816G, D816H, D816I, and mutations such as F522C, V560G, I817V, N819Y, L799F, D820G, N822L, N822I, InsVI815-816, E839K, S840N, and S849I, have also been reported in SM [22,52,53]. *KIT* mutations in the extracellular domain (e.g., deletion of codon 419 in exon 8 or p.A502_Y503dup in exon 9), TM domain (e.g., *KIT* p.F522C), or JM domain (e.g., *KIT* p.V560G) are found more frequently in indolent SM [10]. However, several non-D816V mutations have also been identified in MCL and MCS, such as F522C, V654A, p.A502_Y503dup, and V560G [53]. A high variant allele frequency (VAF) has been reported to be associated with the progression of indolent SM into advanced SM [54]. While children with CM may display *KIT* missense mutations at codon 816, they often harbor alterations in exons 8 or 9 instead [55]. *KIT* mutation is uncommon in WDSM (15%) [56]. Germline *KIT* mutations have been found to be associated with familial mastocytosis [57,58]. A list of *KIT* mutations in mastocytosis (Figure 1, top) from prior published literature was summarized by Valent P. et al. in 2021 [8].

Additionally, other prognostic mutations have been identified in advanced SM. These mutations affect genes encoding epigenetic regulators (*ASXL1*, *DNMT3A*, *EZH2*, and *TET2*), signaling molecules (*CBL*, *JAK2*, *KRAS*, and *NRAS*), transcription factors (*RUNX1*), and splicing factors (*SRSF2*, *SF3B1*, and *U2AF1*) [53]. Most advanced SM patients have ≥2 mutations besides *KIT*. Those lacking additional mutations exhibit a less clinically aggressive course, with less significant cytopenias and significantly longer overall survival (OS) [59]. The presence of mutations in *SRSF2*/*ASXL1*/*RUNX1* (SAR) and/or *EZH2* genes, or *ASXL1*/*CBL*, has been shown to have a significantly adverse prognosis [60,61,62]. The Spanish Network on Mastocytosis group identified pathogenic mutations with VAF (≥30%) in *ASXL1*, *RUNX1*, and/or *DNMT3A* genes as independent predictors for OS in ISM [54].

Cytogenetic abnormalities have been reported to be infrequently encountered (<10%) in patients with ISM and ASM, while they are more common (>25%) in SM-AHN. In ASM, patients with a poor-risk karyotype (complex karyotype and monosomy 7) have a significantly shorter median OS compared to patients with a good-risk karyotype (trisomy 8, 1q-, 5q-, and 12p-) [63,64].

## 4. Clinical Presentations, including Updated B- and C-Findings of SM

The heterogeneous clinical presentation of SM is related to MC-released mediators, MC burden, and associated hematological disorders. Constitutional symptoms, when present, may include weight loss, pain, nausea, headache, malaise, or fatigue [5]. Mediator-associated symptoms occur both in indolent and advanced SM, triggered by factors like stress, allergens, medications, and more [65]. These symptoms, ranging from diarrhea and abdominal pain to skin changes and musculoskeletal issues, can mimic those of other conditions, complicating SM diagnosis [66,67,68].

Extensive involvement with neoplastic MCs is associated with organ dysfunction. Clinical signs and symptoms are classified as B-findings (burden of disease) and C-findings (cytoreduction requiring), which are important factors for establishing an accurate subclassification for SM. B-findings have been slightly modified by the WHO 5th edition and 2022 ICC (Table 3) [9,49]. For BST, the measured level should be adjusted in the setting of known HαT. One suggested approach is to divide BST by the number of extra copies of the *TPSAB1* gene plus one. For example, the corrected BST in a patient with SM and HαT with three additional copies of *TPSAB1* and a measured BST of 400 ng/mL would be 100 ng/mL (400/(3 + 1) = 100) and would thus not qualify as a B-finding [49]. It is noteworthy that the WHO 5th edition considers *KIT* mutation with VAF ≥ 10% in BM cells or peripheral blood leukocytes as a B-finding, while the 2022 ICC does not. Another notable difference is that the 2022 ICC simplified one B-finding to “cytopenia (not meeting criteria for C-findings) or cytosis. Reactive causes are excluded, and criteria for other myeloid neoplasms are not met”.

C-findings represent signs of organ damage caused by neoplastic MC infiltration, reflecting organ dysfunction and disease aggressiveness. The WHO 5th edition has made minor changes to C-findings, whereas the 2022 ICC has kept them the same as in the prior classification (Table 3). It should be noted that osteoporosis and smaller lytic and sclerotic bone lesions are common in all categories of SM and are not considered C-findings [69].

## 5. Updates on Diagnostic Criteria for Systemic Mastocytosis (SM)

The diagnosis of SM is based on the integration of clinical, morphological, and immunophenotypic criteria, often combined with molecular studies. The basic requirement of one major and one minor criterion or three minor criteria for the diagnosis of SM are retained by the WHO 5th edition guidelines. In contrast, the 2022 ICC mandates only 1 major criterion or 3 minor criteria (Table 4). For both classification systems, the major criterion remains the demonstration of multifocal dense infiltrates of MCs (≥15 mast cells in aggregates) in BM or other extracutaneous organ(s). Minor criteria are utilized to confirm elevated MC mediator release, abnormal morphology, clonality, or an aberrant MC phenotype. Notably, the minor immunophenotypic and genetic criteria have been revised in both guidelines [10,50]. First, the expression of CD30 and the presence of any *KIT* mutation causing ligand-independent activation (refer to Figure 1 for reported *KIT* mutations) are now considered minor diagnostic criteria. Second, the adjustment of BST in cases of HαT is specifically highlighted in the WHO 5th edition.

Well-differentiated systemic mastocytosis (WDSM) is rare, accounting for about 5% of MC neoplasms. It is not officially categorized as a variant in the WHO 5th edition classification; instead, it is considered a morphological variation that may occur in any SM subtype, including MCL. Clinically, WDSM presents with an early onset, often in childhood, with a tendency for familial aggregation and a higher prevalence among females. It is characterized by cutaneous involvement [25]. Morphologically, WDSM is identified by BM infiltration by mature-appearing, round and well-granulated MCs, rather than spindle-shaped forms [56]. Typically, MCs in WDSM lack CD2 and CD25 but rather show CD30 expression. The classical *KIT* D816V mutation and other exon 17 *KIT* mutations are typically absent in WDSM; however, mutations in the JM (e.g., V560G and K509I) or TM (e.g., F522C) domains of *KIT* (exons 10–11) and wild-type *KIT* have been reported. These non-D816 *KIT* variants potentially make WDSM more responsive to treatment with imatinib [58].

Diagnosis of WDSM can be challenging due to the well-differentiated morphology, frequent wild-type *KIT,* and low serum tryptase levels. Aberrant CD30 expression appears to be a recurrent and reliable immunohistochemical marker for diagnosing WDSM. Proposed diagnostic criteria for WDSM include fulfilling at least the below major criterion plus one minor criterion, or alternatively meeting three minor criteria in the absence of the major criterion [56]. Like typical SM, the presence of compact MC aggregates in BM sections or smears constitutes the major criterion. Minor criteria include the following: (1) clustering of MCs in pairs or triplets outside of marrow particles on BM aspirate smears; (2) aberrant expression of CD30 and/or overexpression of cytoplasmic proteases (carboxypeptidase A, tryptase, or both; assessed by flow cytometry); (3) identification of *KIT* mutation; and (4) female patient with symptoms dating to childhood or with familial aggregation.

Notably, SM with hypereosinophilia is excluded if there are positive findings for *PDGFRA*, *PDGFRB*, *FGFR1*, *JAK2*::*PCM1*, *FLT3* fusion, or *ETV6*::*ABL1* according to the WHO 5th edition [10]. In rare cases where the tumor exhibits both a tyrosine kinase fusion gene and a *KIT* mutation, the disorder is more accurately categorized as SM-AMN following the guidelines of the 2022 ICC. Testing for these tyrosine kinase gene rearrangements is crucial because morphologically and immunophenotypically abnormal MC populations (with aberrant CD2 and/or CD25 expression) are often encountered in these neoplasms [25]. Similarly, acute myeloid leukemia with t(8;21) may demonstrate both MC expansion and/or *KIT* mutation. However, only a subset of cases fulfills the criteria for SM-AHN [70].

## 6. Updates on Classification for SM Subtypes

### 6.1. Indolent Systemic Mastocytosis (ISM)

Indolent systemic mastocytosis (ISM) is the most common form of SM. It is defined by meeting the criteria for SM, no or only one B-finding, no evidence of C-findings, and no evidence of MCL or associated hematological neoplasm (AHN) [71]. To diagnose ISM without skin lesions, the WHO 5th edition requires a BST level ≥125 ng/mL and/or dense MC infiltrates in an extramedullary organ [49]. Compared to patients with advanced SM, ISM patients typically present at a younger age (median age of 49 years) and exhibit a higher prevalence of UP-like skin lesions, GI symptoms, and a low MC burden (usually <5–10% of BM cellularity). They also have a lower incidence of constitutional symptoms and hepatomegaly (less than 20%) [50,72]. Insect stings, especially from Hymenoptera, are the leading cause of acute MC mediator release episodes in patients with ISM without skin lesions [71,73,74,75,76,77]. ISM generally follows a stable clinical course or progresses slowly, and the overall survival rate for ISM patients is similar to that of the age- and sex-matched US population [78,79].

### 6.2. Bone Marrow Mastocytosis (BMM)

Bone marrow mastocytosis (BMM) is defined as neoplastic MC proliferation solely involving the BM [73]. BMM is characterized by limited BM infiltration, absence of cutaneous lesions, normal or minimally elevated serum tryptase levels, older age, and male predominance [80]. The EU/USA consensus group proposed adjusted diagnostic criteria for BMM in 2021, including fulfillment of SM criteria, serum tryptase (BST) <125 ng/mL, absence of skin lesions, B- or C-findings, lack of dense SM infiltrates in an extramedullary organ, and exclusion of MCL or AHN [8]. In the 2022 ICC, BMM is classified as a subvariant of ISM, whereas the WHO 5th edition delineates BMM as a distinct variant of SM. The WHO 5th edition diagnostic criteria for BMM require meeting SM criteria with low MC burden restricted to BM, no B findings, and a BST level <125 ng/mL [49]. The basis for this distinction is that indolent SM patients without any of these risk factors have better progression-free survival and OS rates than other indolent SM variants [25,81,82]. Patients should receive a diagnosis of ISM without skin lesions, rather than BMM, if any of the following criteria are present: one B-finding, serum tryptase is ≥125 ng/mL, or a dense atypical MC infiltrate is present in an extramedullary organ [8].

BMM patients are often asymptomatic, and the diagnosis may remain unknown or be established only after several years. BMM patients, who are older and predominantly male, exhibit fewer mediator-related symptoms (except for anaphylaxis), normal or slightly increased tryptase levels, and a strong association with severe allergic reactions triggered by Hymenoptera stings [73,80]. BMM shows a significant correlation with severe anaphylaxis, mainly due to an IgE-mediated allergy triggered by Hymenoptera stings [75], and less frequently, to unexplained (idiopathic) anaphylaxis [76]. Although higher mediator levels are strongly correlated with increased BM MC burden, there is no notable link between MC mediator symptoms and mediator levels [71]. Osteoporosis is frequently associated with BMM and might present as the only symptom of the disease [80]. Due to its indolent nature, BMM can be easily overlooked and requires a high degree of clinical suspicion for diagnosis [71,73]. In the absence of skin lesions, BMM should be considered in the differential diagnosis for diverse clinical conditions including unexplained anaphylaxis, Hymenoptera venom allergy, recurrent mediator-related syndromes, and idiopathic osteoporosis, especially in male patients [76], and serum tryptase level should be included for work-up of these conditions [80]. Highlighting its indolent nature, the 10-year progression-free survival for BMM is 95.9% [83]. 

### 6.3. Smoldering Systemic Mastocytosis (SSM)

Smoldering systemic mastocytosis (SSM) is defined by meeting the SM criteria, having ≥2 B-findings, and no C-findings or AHN. SSM is characterized by a relatively high MC burden in the bone marrow (>30%), older age, a higher prevalence of palpable hepatosplenomegaly, and a higher incidence of constitutional syndromes [84]. Relative to other indolent SM subtypes, SSM is associated with inferior OS and a risk of progression to ASM [71,84]. A recent study indicates that survival prediction in SSM appears to depend more on age and the presence or absence of risk factors, rather than MC burden [84].

### 6.4. Aggressive Systemic Mastocytosis (ASM)

**Figure 2:**
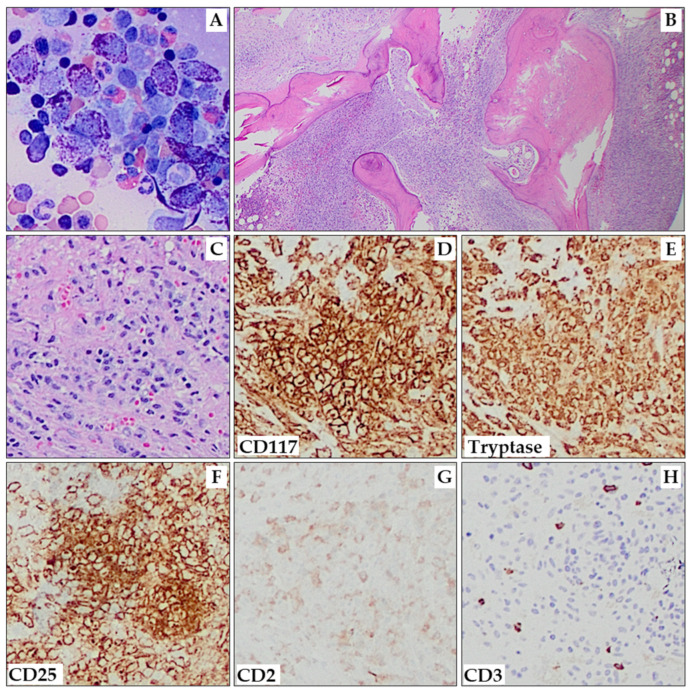
Aggressive systemic mastocytosis. Aspirate smears show areas with numerous hypogranular and/or spindle-shaped MCs (**A**; 200×). The marrow shows multifocal interstitial and paratrabecular aggregates of MCs, as well as a few small areas of residual trilineage hematopoiesis (**B**; 20×). One highlighted MC aggregate (**C**) shows expression of CD117 (**D**), tryptase (**E**), CD25 (**F**), and CD2 (weak, partial; (**G**)). CD3 (**H**) is negative in MCs. (**C**–**H**) are all at 100× magnification.

The diagnosis of aggressive systemic mastocytosis (ASM) requires fulfilling the SM criteria and the presence of ≥1 C-finding. It is important to note that the definition of C-findings has been revised in the WHO 5th edition classification. ASM typically exhibits a high MC burden in the BM (up to 80%), elevated tryptase levels, and is almost always associated with *KIT* D816V mutation [85]. Cutaneous lesions are frequently absent. The presence of atypical type II MCs and metachromatic blasts (>5%) in the bone marrow smear has been associated with shorter survival [85]. ASM in transformation to MCL (ASM-t) is defined as ASM with rapid progression and 5%-19% MCs in BM smears [49,86]. The overall median survival in ASM is 41 months [85]. Figure 2 depicts bone marrow with extensive marrow replacement by ASM, including over 10% mast cells on aspirate smears.

### 6.5. Systemic Mastocytosis with an Associated Hematologic (WHO 5th Edition)/Myeloid (2022 ICC) Neoplasm (SM-AHN/AMN)

SM-AHN is the second most common SM subgroup [87]. By the WHO 5th edition classification, this diagnosis must satisfy criteria both for SM and for another WHO-defined hematologic neoplasm such as myeloproliferative neoplasm (MPN), myelodysplastic syndrome (MDS), or lymphoproliferative disorder. In the 2022 ICC, myeloid neoplasms are the exclusive related hematologic conditions due to their shared *KIT* mutations and clonal abnormalities with SM, distinguishing them from lymphoid neoplasms that co-occur with SM but lack *KIT* mutations [67,68]. The frequent shared clonal origin of AMNs provided the biological rationale for the decision to narrow the scope of this entity in the 2022 ICC. Acquisition of *KIT* D816V may be a late event SM-AHN [88], and *KIT* D816V is usually present when sensitive techniques are applied.

**Figure 3:**
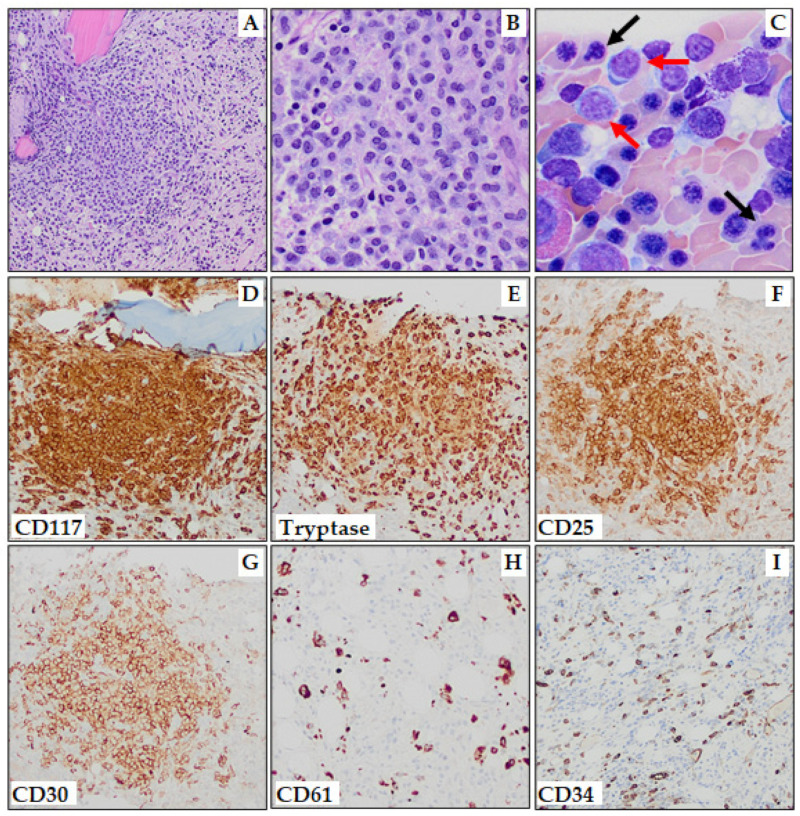
Systemic mastocytosis with an associated myelodysplastic syndrome with increased blasts-1 (SM-MDS-IB-1). (**A**,**B**) One of multiple MC aggregates (**A**, 100×; **B**, 400×). (**C**) Aspirate smear (Wright–Giemsa stain) shows erythroid dysplasia with nuclear budding and irregularity (black arrows) and increased blasts (red arrows). Immunohistochemistry (100×) shows MC aggregates to be positive for: CD117 (**D**), mast cell tryptase (**E**), CD25 (**F**), and CD30 (**G**). CD61 immunostaining highlights dysplastic megakaryocytes (**H**), and CD34 immunostaining highlights ~5% blasts (**I**).

In SM-AHN, both disease components must adhere to WHO definitions, such as SM with associated MDS with increased blasts-1 (Figure 3). Regarding the distribution of AHNs, around 90% are of myeloid lineage, whereas lymphoid neoplasms such as chronic lymphocytic leukemia, lymphomas, multiple myeloma, and primary amyloidosis are rare. Among myeloid neoplasms, chronic myelomonocytic leukemia (CMML) is the most common in SM-AHN. Leukemic transformation occurs more frequently in SM-MDS (29%) compared to SM-MPN (11%) or SM-CMML (6%) [50,72]. If either *PDGFRA*, *PDGFRB*, *FGFR1*, or *JAK2*::*PCM1* gene rearrangement is identified, the diagnosis should be classified as “myeloid or lymphoid neoplasms with eosinophilia and tyrosine kinase gene fusions”; eosinophilia may be a clue to this alternative diagnosis. However, if monocytosis, eosinophilia, splenomegaly, elevated LDH, high *KIT* D816V variant allele frequency, and/or additional somatic mutations in genes associated with myeloid malignancies are identified, it should prompt careful evaluation to exclude SM-AHN. Compared to other subtypes of SM, SM-AHN typically occurs in older individuals, with higher incidences of constitutional symptoms and hematologic abnormalities, leading to an inferior OS [89]. The prognosis of SM-AHN is generally influenced by the aggressiveness of the AHN.

### 6.6. Mast Cell Leukemia (MCL)

**Figure 4:**
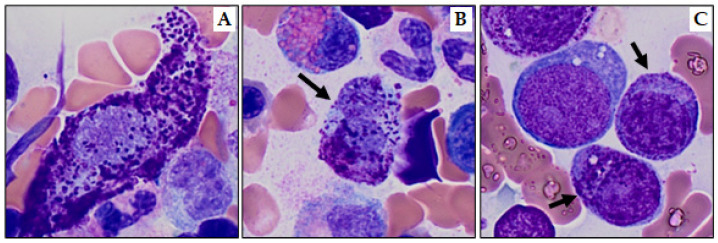
Atypical mast cells (Wright–Giemsa stain, 1000×). (**A**) Spindle shaped (type I) mast cell, (**B**) bilobed promastocyte (type II; black arrow), and (**C**) metachromatic blasts (black arrows).

MCL is an extremely rare and aggressive form of SM, accounting for less than 1% of all mastocytosis cases. It is diagnosed based on meeting SM criteria and the presence of ≥20% atypical MCs in BM aspirate, as specified by both the 5th WHO and the 2022 ICC. It is essential to note that the ≥20% infiltrate refers specifically to the BM aspirate, not the biopsy [69]. Atypical immature MCs include promastocytes (Figure 4B), metachromatic blast-like forms (Figure 4C), or highly pleomorphic MCs [9,33]. According to the 2022 ICC, in cases of suboptimal aspirate (dry tap) MCL can be diagnosed on a BM biopsy showing a dense, diffuse infiltration of atypical immature MCs [9]. Notably, the WHO 5th edition distinguishes the more common aleukemic MCL variant (<10% MCs in peripheral blood) from the “classic” leukemic variant (≥10% circulating MCs), whereas the 2022 ICC does not.

MCL can either be secondary MCL, which occurs as progression from SM, or can present as primary MCL [90]. The WHO 5th edition criteria further subclassifies MCL into acute MCL (with C-findings) and chronic MCL (without C-findings), with the latter having a much better prognosis [49]. However, the entity “chronic MCL” is not recognized by the 2022 ICC [9,50]. Patients with chronic MCL may respond to KIT-targeted therapy and show a slightly improved prognosis [86,91]. However, the overall outcome for MCL, regardless of subtypes, remains poor, with a median survival time ranging from 2 to 31 months [92,93].

## 7. Diagnostic Workup for Systemic Mastocytosis

### 7.1. Tryptase

MCs are the primary source of circulating tryptase. The typical BST level is approximately 5 ng/mL, and any level exceeding 11.4 ng/mL is considered elevated [94]. Increased BST can be seen in many conditions, including HαT, mastocytosis, myeloid neoplasia, MC hyperplasia, and chronic kidney disease. Spurious increases can be caused by assay interference.

During evaluation for MCAS, a BST blood sample should ideally be collected either prior to any MC mediator release event or at least 24 h after resolution of all signs and symptoms [30]. Specimens to confirm tryptase elevation must be obtained within 3 h following a suspected MC activation event to avoid false negative results [26]. The formula of 20% increase in BST plus 2 ng/mL (1.2× BST + 2) is a suggested meaningful indicator of MC activation [1].

In addition to tryptase levels, routine laboratory tests including complete blood count with differential, comprehensive metabolic panel, and routine coagulation parameters, should be included to help identify evidence of organ dysfunction in the diagnostic workup of MC disorders. These tests are also useful to monitor and follow the clinical course of patients with SM.

### 7.2. Morphological Features

In the peripheral blood of patients with SM, anemia and eosinophilia are common. The peripheral blood smear should be reviewed for circulating MCs as well as for evidence of an AHN (dysplasia, circulating blasts, or abnormal monocytes).

BM aspirate may display a low number of MCs due to marrow fibrosis and often yields false-negative results. Abnormal MCs can show variable features, including round or spindled shape, hypogranular cytoplasm or unevenly distributed granules, and oval nuclei with eccentric (decentralized) position, immature chromatin, and/or multi-nucleation. Abnormal MCs can be subdivided into three pathological types and occur in different subtypes of SM [19]: atypical type I (spindled or hypogranular), atypical type II (pro-mastocytes with bi- or multi-lobed nuclei), and metachromatic blast cells (Figure 4A–C). However, an atypical morphology of MCs does not count as SM criterion when identified in or adjacent to BM particles on aspirate smears [50]. A comprehensive assessment of other myeloid cells could reveal indications of an associated MDS or MPN, which are crucial for establishing a diagnosis of SM-AHN. More severe cytologic atypia of MCs may indicate a more aggressive SM variant [9].

On BM core biopsies, MC aggregates can appear paratrabecular, perivascular, or interstitial and are often focal, although they can occasionally be diffuse. These lesions are frequently associated with increased fibrosis and/or osteosclerosis. MC aggregates are often comprised of central cores of lymphoid cells surrounded by atypical MCs (or vice versa), eosinophils and histiocytes. In both the 2022 ICC and the WHO 5th edition, abnormal MC morphology (≥25%) counts as a minor SM criterion. Notably, spindle-shaped MC forms do not meet the SM minor criterion when adjacent to vascular cells, fat cells, nerve cells, or the endosteal-lining cell layer [72]. For WDSM, MCs are round with central nuclei and robust granulation. When assessing abnormal MC morphology, both dense and diffuse MC infiltrates should be examined [95], and the percentage of morphologically abnormal MCs as well as the specific morphological features should be addressed. In rare cases, atypical spindle shaped MCs may diffusely infiltrate BM without significant aggregation [96]. In this setting, despite the absence of the major diagnostic criterion, identification of three minor criteria will still confirm the diagnosis of morphologically occult SM.

### 7.3. Immunophenotypic Features

Immunohistochemical studies for MCs and other cell types are standard procedures in the diagnostic evaluation for both BM and other extramedullary tissues in cases of suspected or confirmed SM. MCs can be detected using tryptase and/or KIT/CD117 as immunohistochemical markers. Occasionally, MCs stain only weakly positive or are negative for tryptase [72,97]. Therefore, KIT/CD117 should always be added to the panel of MC-specific markers, especially when MCs are immature as in advanced SM and in cases of suspected MCS.

Expression of CD2, CD25, and/or CD30 is one of the minor criteria set by both the 2022 ICC and the WHO 5th edition, which should always be evaluated by immunohistochemistry and/or flow cytometry. Importantly, MC flow cytometry should be treated as a rare event analysis, ideally with the acquisition of one million or more events [69]. CD25 is more frequently expressed than CD2 (higher sensitivity) and is additionally the most specific neoplastic mast cell marker [98]. However, there are cases in which MCs express CD25, but SM cannot be diagnosed. Examples include chronic inflammatory reaction, post chemotherapy setting, and myeloid/lymphoid neoplasms associated with *PDGFRA* rearrangement [99]. However, unlike the dense aggregates of MCs seen in the BM of patients with SM, the MCs associated with *FIPIL1::PDGFRA* are typically scattered or form loose clusters and typically show no CD2 expression [100,101]. Both the 2022 ICC and the WHO 5th edition added CD30 expression as a minor SM criterion. This marker is particularly useful for WDSM, which characteristically lacks CD2 and CD25 expression on neoplastic MCs.

Neoplastic MCs also may express CD123, the alpha-subunit of the high affinity receptor for interleukin-3, with differential expression in disease subgroups. For example, in one study, CD123 showed 100% expression in advanced SM, which is often associated with focal proliferation of brighter staining plasmacytoid dendritic cells (PDCs) [102]. CD123 expression and its staining intensity had prognostic value in SM-CMML and non-indolent SM cases. Basophils are positive for CD123 but negative for CD117, which is particularly useful in differentiating MCL from acute basophilic leukemia.

In addition to MC markers (CD117, tryptase, CD2, CD25, and CD30), an extended immunohistochemistry panel may include CD34 (to detect and quantify myeloblasts), CD14 and CD68 (monocytes), CD71 and E-cadherin (erythroid cells), CD138 (plasma cells), CD3 (T cells), CD20 (B cells), and a megakaryocytic marker (CD31, CD42b, or CD61).

### 7.4. Cytogenetic and Molecular Studies

Chromosome analysis and/or FISH should be included in patients with a suspected AHN. While the chromosomal abnormality itself may not be sufficient for a diagnosis, it can contribute to supporting the diagnosis of an AHN, depending on the number of affected metaphases or inter-phases [8,63,64,72]. Cytogenetic studies should be included when disease progression is suspected. To prevent misdiagnosis between SM with hypereosinophilia and myeloid/lymphoid neoplasms with eosinophilia, it is crucial to perform FISH testing for tyrosine kinase fusion genes due the cryptic nature of some the rearrangements.

In both the latest WHO and ICC criteria, the presence of activating *KIT* point mutation(s) at codon 816 or other critical regions of *KIT* is considered a minor SM criterion. The scope of SM-associated *KIT* variants documented in the literature, including confirmed activating mutations that serve as a minor SM criterion, are illustrated in Figure 1. To avoid false-negative results in patients suspected of having mastocytosis with mildly elevated serum tryptase levels or subtle symptoms, utilization of highly sensitive methods like digital droplet PCR (ddPCR) and allele-specific oligonucleotide-based PCR (ASO-qPCR) are recommended to screen for *KIT* D816V mutations in peripheral blood or BM [69,103,104]. When these tests are negative but clinical suspicion is high, next generation sequencing-based mutational analysis of the *KIT* gene should be considered, as this technique can identify alternative D816 and non-D816 codon mutations, albeit with lower sensitivity than ddPCR and ASO-qPCR.

## 8. Updates on Treatment for Systemic Mastocytosis

### 8.1. Non-Advanced SM: ISM, BMM, and SSM

The treatment of ISM and stable SSM focuses primarily on the prevention and treatment of anaphylactic reactions and symptom relief using histamine receptor blockers and MC-stabilizing agents [105,106]. Specific triggers that cause MC mediator release should be avoided. Cytoreductive agents such as cladribine or pegylated interferon alfa-2a alleviate MC activation symptoms by reducing MC burden and directly blocking MC activation, respectively. Although cytoreductive therapy is generally not indicated for indolent SM, it may have a role for treating select patients with severe refractory MCAS or bone disease [78,107,108,109,110].

Among conventional therapies, cromolyn, an inhibitor of MC degranulation, is useful for improving GI, cutaneous, and neurological symptoms [106,111,112,113,114]. Aspirin, corticosteroids, and leukotriene receptor antagonists have proven effective in managing symptoms unresponsive to other treatments [115]. Montelukast, a leukotriene receptor antagonist that blocks the effects of MC-derived leukotrienes, can be applied particularly for persistent headaches, musculoskeletal pain, and flushing [114,116]. Bisphosphonates should be prescribed in cases of marked osteopenia and osteoporosis when the T score is below the recommended level [117]. As for biologics, the IgE specific humanized monoclonal antibody omalizumab has shown particular efficacy against life-threatening anaphylaxis, while also improving skin and GI symptoms [118].

The classic D816V-mutated KIT receptor is not responsive to imatinib [57,119], limiting its use in the treatment of all forms of SM. However, some non-D816 mutations, which are over-represented in WDSM, show sensitivity to imatinib [57]. Masitinib, another tyrosine kinase inhibitor (TKI) with activity against wild-type KIT and JM-activating mutations, but not against KIT D816V, has demonstrated efficacy in alleviating flushing, pruritus, and depression in mastocytosis patients [106]. Midostaurin (PCK412) was shown to be effective in ISM patients with severe symptoms refractory to antihistamines in a phase 2 trial [120]. See below for more details about these targeted therapies.

### 8.2. Advanced SM (ASM, SM-AHN, and MCL)

The treatment of advanced SM (ASM, SM-AHN, and MCL) involves various factors, such as managing symptoms and minimizing organ damage. Addressing MC release-related symptoms in advanced SM follows a similar approach to that of patients with ISM. However, advanced subtypes are characterized by MC infiltration in organs, requiring the use of TKIs and/or cytoreductive agents to control/decrease MC burden. Available therapeutic options beyond TKIs include interferon α, cladribine, and allogeneic stem cell transplant, with transplant employed particularly for selected younger patients. The current NCCN guidelines classify cytoreductive therapy into three categories: preferred regimens (avapritinib and midostaurin), other recommended regimens (cladribine), and those useful under specific circumstances [27].

Interferon (IFN-α) can reduce symptoms related to MC activation and improve cutaneous lesions, BM MC burden, GI and skeletal symptoms, and C-findings in all SM subvariants [121]. Combination with corticosteroids may improve IFN-α efficacy and tolerability [122]. The time to best response may be longer relative to other cytoreductive therapies due to cytostatic action, meaning IFN-α could be more useful for patients with a slow progression who do not need rapid cytoreduction. Major toxicities include fatigue, depression, and thrombocytopenia. Pegylated-interferon α-2a or α-2b is suggested for better tolerability [123]. Pegylated-interferon α-2a ± prednisone is listed as an “other recommended regimen” by NCCN for patients with ASM and SM-AHN [27]. Cladribine (2CdA) reduces MC activation symptoms and C-findings by improving MC burden and should be considered for patients requiring rapid debulking of MC burden, though it is not FDA-approved for SM [124,125,126]. One major toxicity associated with cladribine is myelosuppression; it is also contraindicated in pregnancy.

Imatinib gained FDA approval in 2006 for adult patients with ASM lacking the *KIT* D816V mutation or with unknown *KIT* mutational status. Ideally, its use can be rationally selected for patients with imatinib-responsive *KIT* mutations such as F522C, V560G, germline K509I, deletion of codon 419 in exon 8, and A502_Y503dup in exon 9 [27]. This personalized implementation of imatinib includes patients with WDSM, especially where non-exon 17 *KIT* mutations are common [57,78]. Notably, imatinib demonstrates high efficacy in treating eosinophilia associated with *FIP1L1::PDGFRA* that may have increased MCs resembling mastocytosis [127].

Midostaurin (PCK412) effectively inhibits both wild-type and D816V-mutated *KIT* and is FDA approved for the treatment of advanced SM [128]. Its clinical response rates in ASM are similar regardless of subtype, *KIT* mutation status (*KIT* D816V+, *KIT* D816V-negative, or unknown mutation status), or prior therapy exposure [129]. However, not all patients with advanced SM respond to midostaurin, and acquisition or increasing VAF in *K*/*NRAS*, *RUNX1*, *IDH2*, or *NPM1* genes were associated with disease progression. A recent small phase 2 trial additionally demonstrated the efficacy of midostaurin for adult, *KIT* D816V mutant ISM patients with severe MCAS refractory to antihistamines [120].

Avapritinib (BLU-285, Ayvakit, Blueprint Medicines Corporation, Cambridge, MA, USA), a potent, highly selective inhibitor of KIT p.D816V, has been approved by the FDA for advanced SM (including ASM, SM-AHN, and MCL). Avapritinib treatment was associated with a marked reduction in symptoms and marrow MC burden, decreased tryptase level, spleen volume and *KIT* D816V VAF [130,131,132,133], and improved overall survival compared to midostaurin and cladribine [133]. The most common side effects are myelosuppression causing cytopenias, peripheral edema, periorbital edema, and fatigue [132].

Allogeneic hematopoietic stem cell transplantation (AlloHSCT) should be considered in eligible ASM patients with a suitable donor following successful debulking. For a consensus on indication for alloHSCT in patients with advanced SM, see [134]. Patients with ASM or SM-AHN have better outcomes with alloHSCT compared to those with MCL [135]. The role of alloHSCT and the optimal conditioning regimens require further clarification, and additional clinical trials may provide valuable insight.

Given the identification of frequent aberrant CD30 expression by neoplastic MCs in SM, brentuximab vedotin (BV), a CD30-directed antibody–drug conjugate, is currently being investigated in advanced SM [136,137]. A phase 2 clinical trial in patients with CD30+ ASM and MCL showed that BV was well-tolerated but did not show improvement of MC burden or symptoms; thus, it is not recommended to use BV as monotherapy in ASM patients [138]. Its validity as a component of combination therapy remains to be determined. Other clinical trials are ongoing for potential targeted therapies including anti-CD52, anti-CD33, and anti-PDL1 agents [139].

## 9. Conclusions

Disorders of MCs range from reactive expansions to pathologic hyperactivity to clonal proliferations, with a broad range of associated symptoms and varied natural histories. An increase in normal MCs results in MC hyperplasia. MCAS describes a syndrome associated with chronic MC activation and excessive mediator release, with clinical courses ranging from indolent with normal life expectancy to highly aggressive with reduced survival times. Mastocytosis encompasses the pathologic accumulation of abnormal, clonal MCs in different organs, mostly driven by *KIT* mutations, that causes local and systemic symptoms through tissue infiltration and MC mediator release. It can present as CM, SM, and MCS, with SM including both indolent and advanced subtypes.

The diagnosis of SM requires the synthesis of BM morphologic, immunophenotypic, and molecular (especially next generation sequencing) findings (*KIT* mutation), as well as clinical signs and symptoms (B-findings and C-findings). The 2022 ICC and WHO 5th edition classification define the diagnosis in detail, with subtle differences existing between the two schemas.

Indolent SM, although producing morbidity, is associated with normal OS. In contrast, patients with advanced SM typically have a much-shortened life expectancy in addition to a decreased quality of life. The classical driver *KIT* D816V mutation is a hallmark of and minor diagnostic criterion for SM; other *KIT* mutations have also been reported including germline *KIT* mutations associated with familial mastocytosis. Additional recurrent mutations in SM offer prognostic information and may support a diagnosis of SM-AHN [27]. The complex integrated diagnostic process for SM is complicated by pitfalls and requires expertise to avoid misdiagnosis.

All MC disorders may benefit from therapies to manage symptoms caused by MC-mediated symptoms and organ dysfunction. Additionally, advancements in understanding the biology of neoplastic MCs have led to improved therapeutic approaches targeting the genetic drivers of the disease. Continuing characterization of the phenotypic and molecular architecture of mastocytosis will enable future progress in combatting this rare disease. For example, the observation of CD123 expression by neoplastic MCs in advanced SM offers rationale for therapeutic targeting of this surface molecule in select cases.

## Figures and Tables

**Table 1:** Variants of MCAS and diagnostic features (Adopted from [30,33]).

Variants of MCAS	Diagnostic Features
Primary MCAS (Monoclonal MCAS)	*KIT* D816V+; MCs aberrantly express CD25 in most cases, may either fulfill criteria for mastocytosis (SM or CM) or present with only two minor SM criteria.
Secondary MCAS	IgE-mediated allergy, another hypersensitivity reaction, or another immunologic disease induces MC activation; no neoplastic MCs or *KIT* D816V.
HαT MCAS	HαT is detected, all diagnostic MCAS criteria are fulfilled, and no related allergic cause or underlying clonal MC disease is detected.
Combined MCAS	Patients with MCAS suffering from two or more of the following: (a) CM or SM; (b) overt allergy/atopic disease; (c) a known genetic predisposition such as HαT.
Idiopathic MCAS	MCAS criteria are met, but no related reactive disease, no IgE-dependent allergy, and no neoplastic/clonal MCs.

CM = cutaneous mastocytosis, SM = systemic mastocytosis.

**Table 2:** Types and subtypes of mastocytosis per 2022 ICC [9,33] and WHO 5th edition ^#^ [10,49].

	2022 ICC	WHO 5th Edition
CM	Maculopapular CM (MPCM; previously known as urticaria pigmentosa) Monomorphic Polymorphic	Urticaria pigmentosa/Maculopapular CM Monomorphic Polymorphic
	Diffuse CM (DCM)	Diffuse CM
	Mastocytoma of the skin *	Cutaneous mastocytoma Isolated mastocytoma Multilocalized mastocytoma
SM	Indolent SM (Includes bone marrow mastocytosis **)	Bone marrow mastocytosis **
		Indolent SM
	Smoldering SM	Smoldering SM
	Aggressive SM	Aggressive SM
	SM with an associated myeloid neoplasm	SM with an associated hematologic neoplasm
	Mast cell leukemia	Mast cell leukemia
MCS	Mast cell sarcoma	Mast cell sarcoma

^#^ Well-differentiated systemic mastocytosis (WDSM) represents a morphologic variant that may occur in any SM type/subtype, including mast cell leukemia [49,50]. * In case of ≥ 4 lesions, a diagnosis of MPCM is favored over mastocytoma by 2022 ICC [33]. ** The 5th WHO recognizes BMM as an independent SM subcategory, while the 2022 ICC considers BMM a variant of indolent SM (ISM).

**Table 3:** B- and C-findings by the 2022 ICC and the 5th WHO (Modified) [9,10,49].

	2022 ICC [9]	WHO 5th Edition [10,49]
B-findings	High MC burden (>30% of BM cellularity by MC aggregates, as assessed on BM biopsy) and serum tryptase >200 ng/mL.	High MC burden: Infiltration grade (MC) in BM ≥30% in histology (IHC) and/or serum tryptase ≥200 ng/mL and/or *KIT* D816V VAF ≥10% in BM or PB leukocytes.
	Cytopenia (not meeting criteria for C findings) or -cytosis. Reactive causes are excluded, and criteria for other myeloid neoplasms are not met.	Signs of myeloproliferative and/or myelodysplasia: hypercellular BM with loss of fat cells and prominent myelopoiesis ± left shift and eosinophilia ± leukocytosis and eosinophilia and/or discrete signs of myelodysplasia (<10% neutrophils, erythrocytes, and megakaryocytes).
	Hepatomegaly without impairment of liver function, or splenomegaly without features of hypersplenism including thrombocytopenia, and/or lymphadenopathy (>1 cm size) on palpation or imaging.	Organomegaly: palpable hepatomegaly without ascites or other signs of organ damage and/or palpable splenomegaly without hypersplenism and without weight loss and/or palpable lymphadenopathy or visceral lymph node enlargement found by imaging (>2 cm).
C-findings *	BM dysfunction manifested by one or more cytopenia(s): ANC <1 × 10^9^/L, Hb <10 g/dL, PLT <100 × 10^9^/L, but no obvious non-MC hematopoietic malignancy.	One or more cytopenia(s): ANC <1 × 10^9^/L, Hb <10 g/dL, or PLT <100 × 10^9^/L.
	Palpable hepatomegaly with impairment of liver function, ascites and/or portal hypertension.	Hepatopathy: ascites and elevated liver enzymes ± hepatomegaly or cirrhotic liver ± portal hypertension.
	Palpable splenomegaly with hypersplenism.	Spleen: Palpable splenomegaly with hypersplenism ± weight loss ± hypalbuminemia
	Malabsorption with weight loss due to GI MC infiltrates.	GI tract: malabsorption with hypoalbuminemia ± weight loss.
	Skeletal involvement with large osteolytic lesions and/or pathological fractures.	Bone: large-sized osteolysis (≥2 cm) with pathologic fracture ± bone pain.

* C-findings in 2022 ICC has no changes, adopted from prior 2016 WHO classification [7]. ANC, absolute neutrophil count; BM, bone marrow; Hb, hemoglobin; PB, peripheral blood; PLT, platelets.

**Table 4:** Diagnostic criteria for SM by 2022 ICC and the WHO 5th Edition (Adopted from [9,10,49]).

	2022 ICC	WHO 5th Edition
Major criterion	Multifocal dense infiltrates of tryptase- and/or CD117 positive MCs (≥15 MCs in aggregates) detected in sections of bone marrow and/or other extracutaneous organ(s).	Multifocal dense infiltrates of MCs (≥15 MCs in aggregates) in bone marrow biopsies and/or in sections of other extracutaneous organ(s).
Minor criteria	In bone marrow biopsy or in section of other extracutaneous organs, >25% of MCs are spindle shaped or have an atypical immature morphology.	At least 25% of all MCs are atypical cells (type I or type II) on bone marrow smears or are spindle-shaped in MC infiltrates detected in sections of bone marrow or other extracutaneous organs.
	*KIT* D816V mutation or other activating *KIT* mutation detected in bone marrow, peripheral blood, or other extracutaneous organs.	Activating *KIT* point mutation(s) at codon 816 or in other critical regions of *KIT* in bone marrow or another extracutaneous organ(s).
	MCs in bone marrow, peripheral blood, or other extracutaneous organs express CD25, CD2, and/or CD30, in addition to MC markers.	MCs in bone marrow, blood, or another extracutaneous organs express one or more of the following: CD2 and/or CD25 and/or CD30.
	Elevated serum tryptase level, persistently >20 ng/mL. In cases of SM-AMN, an elevated tryptase does not count as an SM minor criterion.	Baseline serum tryptase concentration > 20 ng/mL (in the case of an unrelated myeloid neoplasm, an elevated tryptase does not count as an SM criterion. In the case of a known HαT, the tryptase level should be adjusted).
**NOTE:**	**The major criterion alone is enough, or in the absence of the major criterion, at least 3 of the 4 minor criteria must be present.**	**The major plus at least 1 minor, or 3 minor criteria must be fulfilled for diagnosis of SM.**
